# Supplementary material for: 5-ALA Is a Potent Lactate Dehydrogenase Inhibitor but Not a Substrate: Implications for Cell Glycolysis and New Avenues in 5-ALA-Mediated Anticancer Action
Source: Cancers (Basel). 2022 Aug 18;14(16):4003. doi: 10.3390/cancers14164003 (PMC9406570; doi:10.3390/cancers14164003)
Supplement: Supplementary file 1 [file cancers-14-04003-s001.zip › cancers-1846903-supplementary.pdf]

SUPLEMENTARY MATERIAL

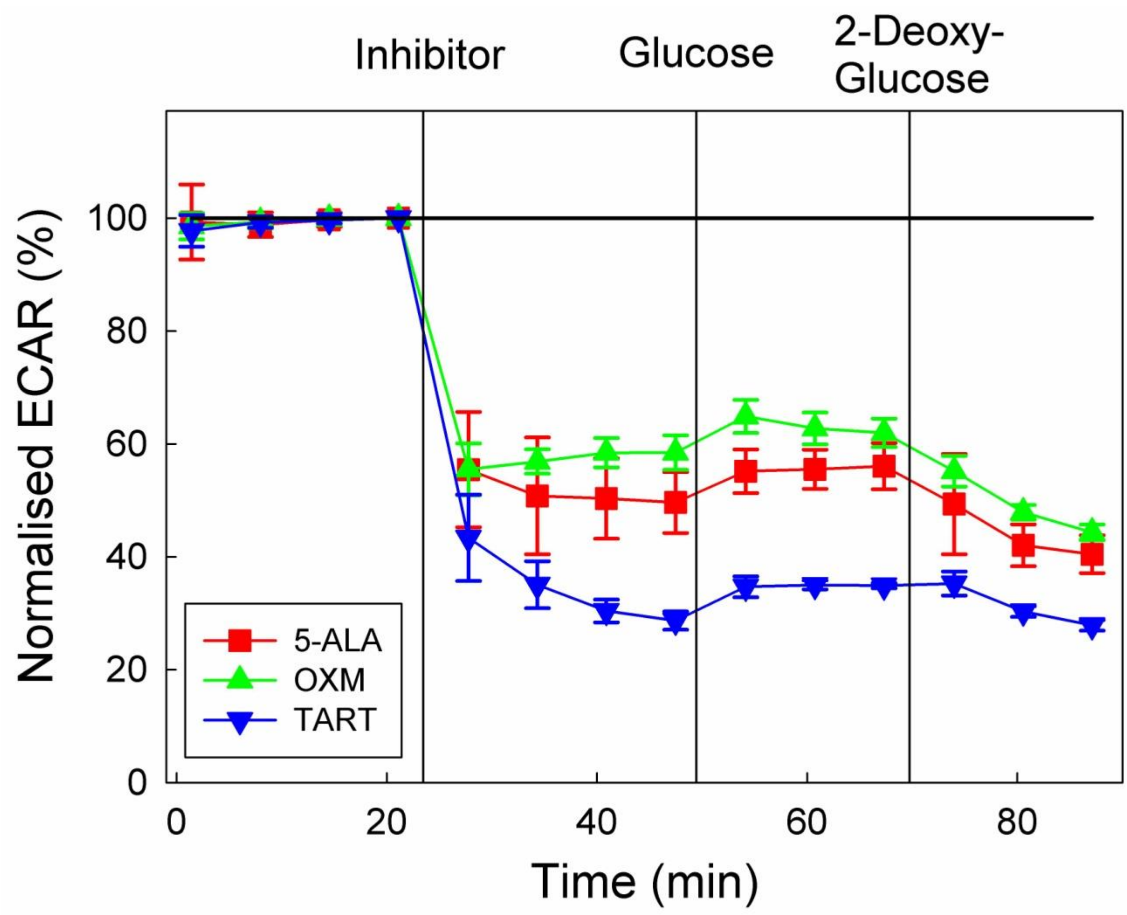

Figure S1. Drop in the glycolytic activity of M059K cells upon addition of 5-ALA, oxamic acid or tarronic acid (1000 μM). Error bars represent one SD.

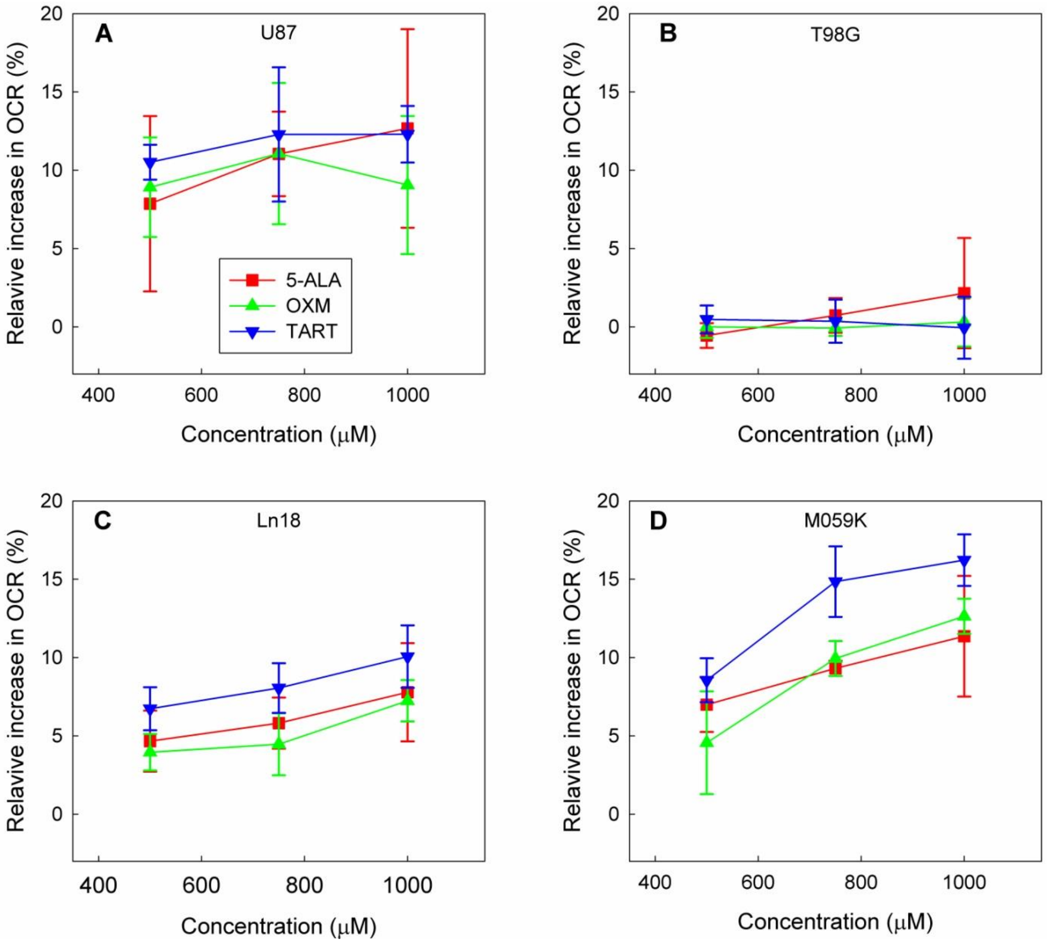

Figure S2. Increase in OCR, following the addition of LDH inhibitors 5-ALA, oxamic acid, tartronic acid. Error bars represent one SD.

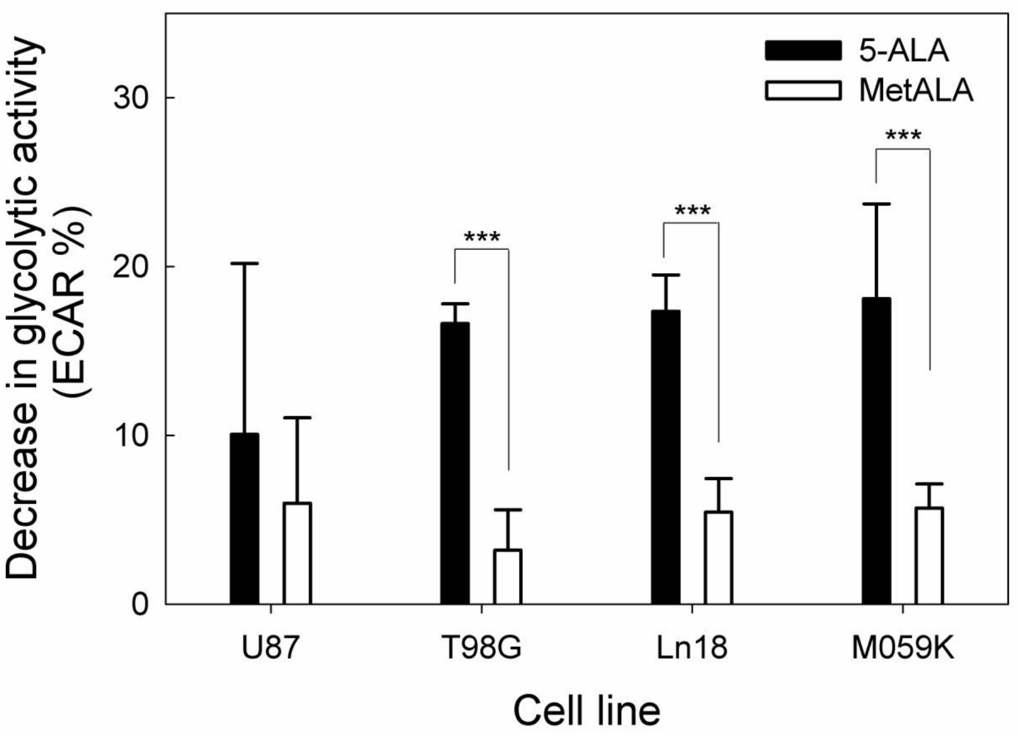

Figure S3. Decrease in glycolytic activity of GBM cell lines upon addition of 1000 μM 5-ALA (black columns) or MetALA (white columns). Error bars represent one SD. *** $P < 0.001$

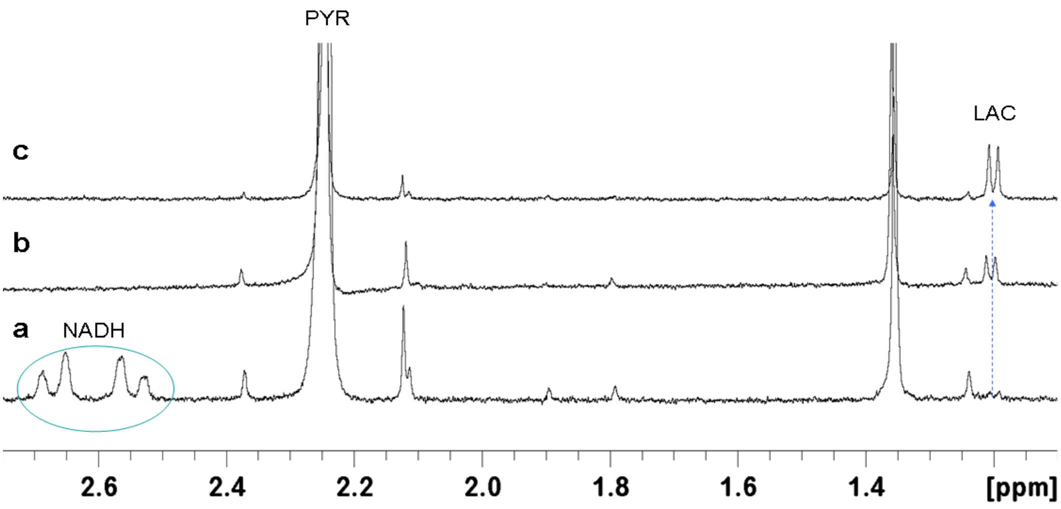

Figure S4. Partial ^1H NMR spectra (500 MHz, 296 K) in phosphate buffer in H_2O, pH 6.51 with external D_2O lock and presaturation of the residual solvent peak: a) sodium pyruvate (20 mM) and LDH (5 μM), b) + NADH (0.4 mg/mL, 0.56 mM), c) + NADH (0.8 mg/mL, 1.12 mM). PYR = pyuvate; LAC = lactate. Increased production of lactate and full consumption of added NADH is observed in b and c.

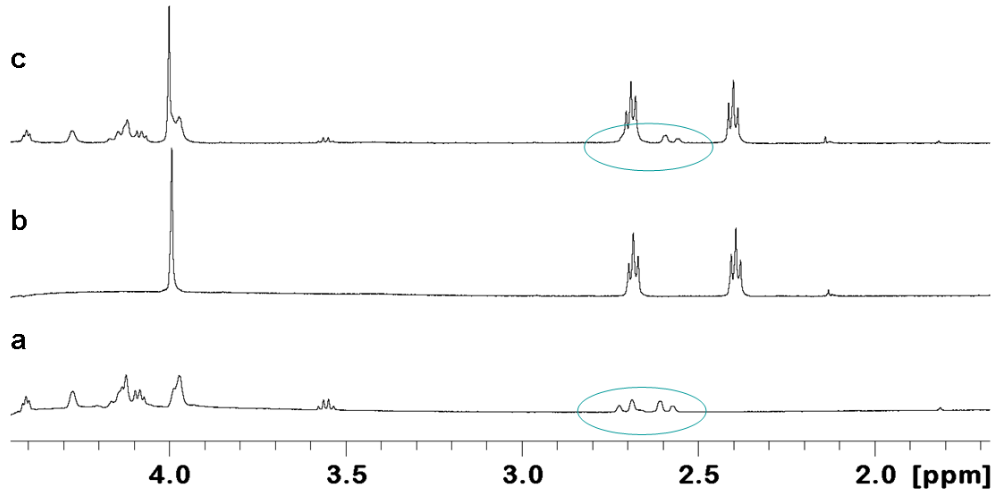

Figure S5. Partial ^{1}H NMR spectra (500 MHz, 296 K) in phosphate buffer in H_2O pH 6.51 with external D_2O lock and presaturation of the residual solvent peak: a) NADH alone, b) 5-ALA.HCl (5 mM) mixed with LDH (10 µM) c) 5-ALA.HCl (5 mM) mixed with LDH (10 µL/mL) and NADH (1.57 mM) monitored for 70 min. No change of signals or emergence of new signals is observed.

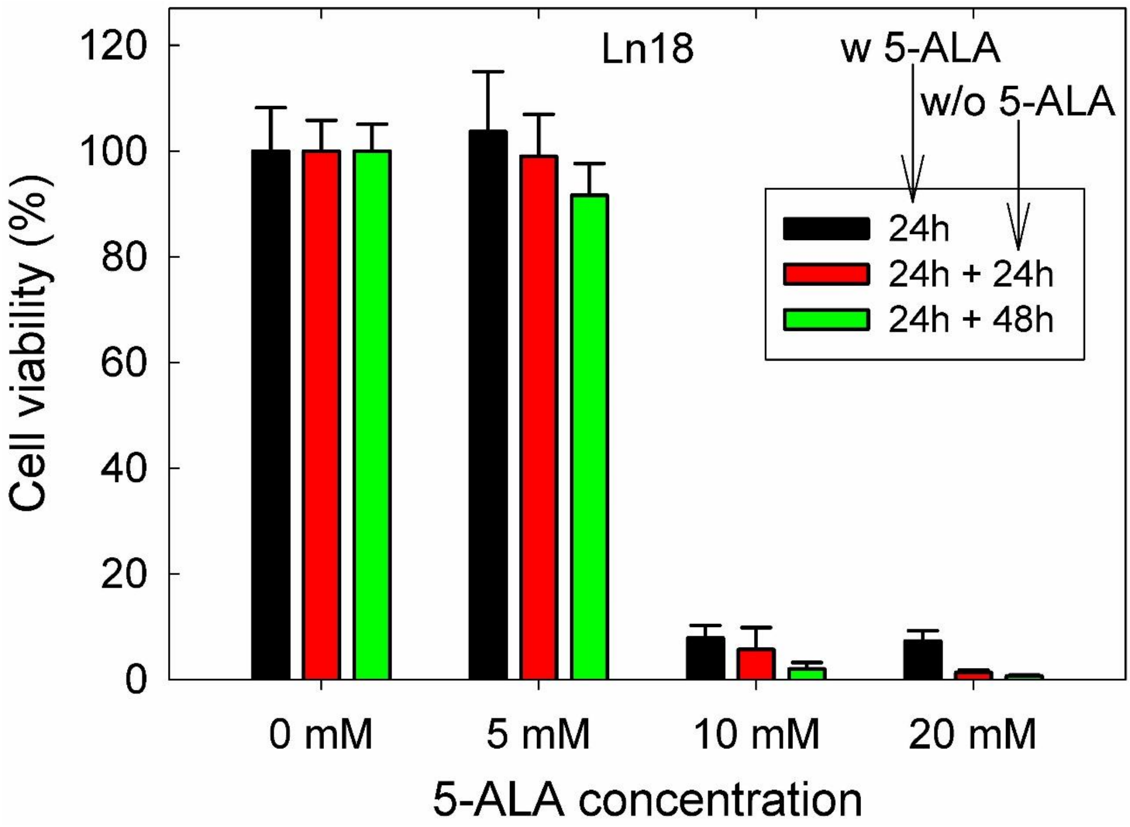

Figure S6. Cytotoxic effects of 5-ÁLA-induced glycolysis inhibition on the LN18 GBM cell line. The viability was in all cases assessed by a standard MTT assay. Cell viabilities are reported following 24h incubation with 5-ALA and an extra 24 and 48h with the 5-ALA removed. Error bars represent one SD.

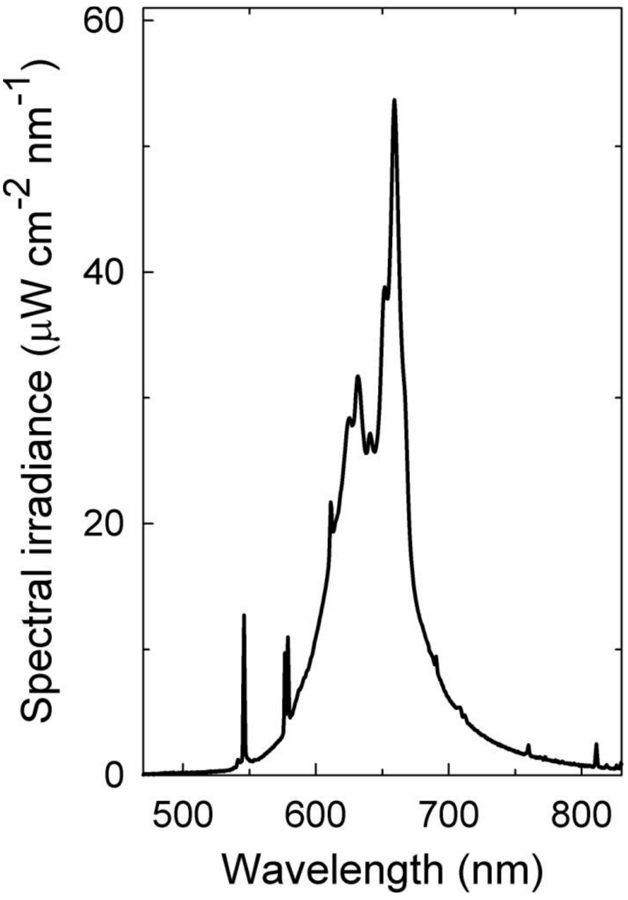

Figure S7. Spectrum of the lamp used for the 5-ALA -PDT
